# Goos-Hänchen shift in cryogenic defect photonic crystals composed of superconductor HgBa_2_Ca_2_Cu_3_O_8+δ_

**DOI:** 10.1371/journal.pone.0302142

**Published:** 2024-05-09

**Authors:** Fangmei Liu, Haiyang Hu, Dong Zhao, Fanghua Liu, Miaomiao Zhao

**Affiliations:** 1 School of Electronic and Information Engineering, Hubei University of Science and Technology, Xianning, China; 2 Laboratory of Optoelectronic Information and Intelligent Control, Hubei University of Science and Technology, Xianning, China; Chulalongkorn University Faculty of Engineering, THAILAND

## Abstract

We explore theoretically Goos-Hänchen (GH) shift around the defect mode in superconducting defective photonic crystals (PCs) in cryogenic environment. The defective PCs are constructed by alternating semiconductors and superconductors. A defect mode arises in the photonic bandgap and sensitively depends on environment temperature and hydrostatic pressure. Reflection and transmission coefficient phases make an abruptly jump at the defect mode and giant GH shifts have been achieved around this mode. The maximum GH shift can get as high as 10^3^*λ* (incident wavelength), which could be modulated by the values of temperature and hydrostatic pressure. This study may be utilized for pressure- or temperature-sensors in cryogenic environment.

## 1. Introduction

Goos-Hänchen (GH) shift arises from angular dispersion of a reflected light beam which is composed of different wave components in a light beam [1–3]. GH shift could be divided two types, *viz*. spatial and angular GH shifts. Numerous studies have been demonstrated that giant GH shifts existed in optical system experimentally and GH shift can be utilized for highly sensitive sensors [4–6]. However, GH shift is several or tens times of the incident wavelength, therefore, it is quite difficult to observe GH shift in practice [7]. To improve the magnitude of GH shift, numerous projects, such as weak loss dielectrics, non-Hermitian systems and graphene composite structures, have been proposed recently [8–10].

The previous investigations in GH shift were all discussed at room temperature, while the studies in low temperature environment are hardly reported. It is well known that the optical and electronic properties of superconductors and semiconductors are extremely sensitive to temperature and hydrostatic pressure [11–14]. The refractive indices of superconductors and semiconductors are functions of these external parameters. A great number of research results show that the optial properties of photonic bandgap, defect mode and Fano resonance in photonic crystals highly are dependent on environment temperature and hydrostatic pressure [15–17]. Especially, superconductors are lossless as environment temperature below the critical value for DC signals, while semiconductors present weak loss characteristics. Otherwise, giant GH shift may be achieved around defect modes of photonic crystals [18]. Therefore, it is significant to investigate the GH shift of low temperature in defective photonic crystals composed of superconductors and semiconductors.

One-dimensional defective superconducting periodic photonic crystals are here constructed and the defect locates at the center. Dependence of the photonic bandgap and the defect mode in bandgap on temperature and hydrostatic pressure are explored. Then, we demonstrate the reflection coefficient phase abrupt change around the defect mode and giant GH shift of reflected beams induced. Furthermore, the influence of environment temperature and hydrostatic pressure on the characteristics is discussed as well. The study may have a potential of application in cryogenic temperature- or pressure-optics sensors.

## 2. Defect superconductor photonic crystals

**Fig 1:**
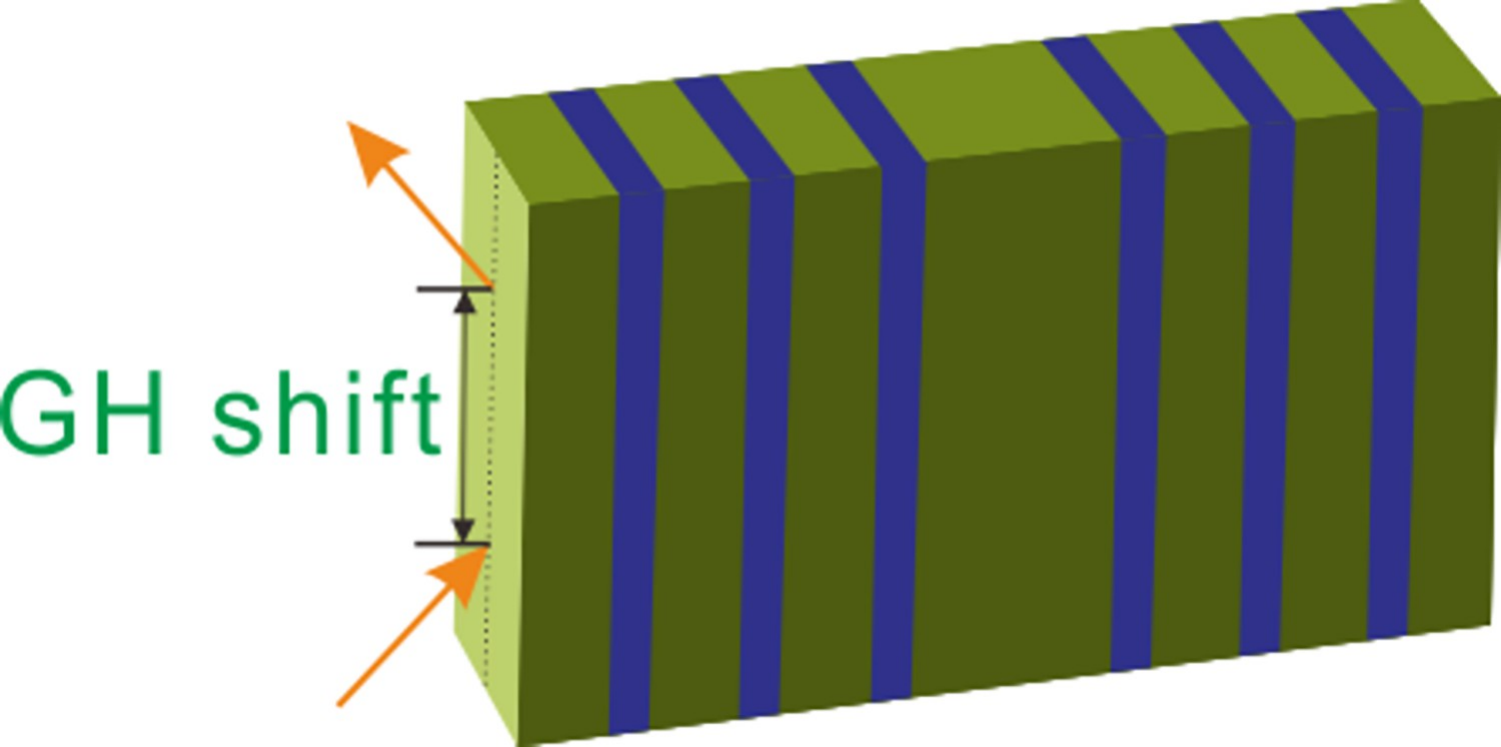
Schematic of GH shift in defect superconductor PC.

The complex system is constructed by alternative lattice elements of superconductor A and semiconductor B given by Fig 1. The whole structure can be described briefly as (AB)^*N*^AA(BA)^*N*^, where *N* = 1, 2, 3, … is the periodic number of PCs. Here we take *N* = 3 to demonstrate the optical properties of system, so the whole photonic crystal can be denoted by ABABABAABABABA. One can see that the system equates to a defect truncation PCs and is symmetric to the central point.

The superconductor lattice element of A is set as HgBa_2_Ca_2_Cu_3_O_8+δ_, which dielectric constant can be ruled by the law of

$$\varepsilon_{a}(\omega )=1-\frac{c^{2}}{\omega^{2}\lambda_{L}^{2}},$$ (1)

where the symbol of c represents the speed of light in vacuum, *λ*_*L*_ is the London penetration depth of the superconducting material, the angular frequency of light waves is given by ω = 2πc/*λ* and *λ* is the wavelength of incident light waves [19]. The value of the London penetration depth of HgBa_2_Ca_2_Cu_3_O_8+δ_ is governed by this expression

$$\lambda_{L}(T_{e})=\frac{\lambda_{0}}{{\left[1-{\left(\frac{T_{e}}{T_{c}}\right)}^{3}\right]}^{1/3}},$$ (2)

where the label of *T*_e_ represents circumstance temperature and the critical temperature *T*_c_ can be regulated by hydrostatic pressure *P*, *viz*. *T*_c_ = A_1_ + B_1_*P* + C_1_*P*^2^. The relevant parameters are provided by A_1_ = 134, B_1_ = 2.009, and C_1_ = −4.194×10^−2^ [14]. HgBa_2_Ca_2_Cu_3_O_8+δ_ is a typically high temperature superconductor with a critical temperature *T*_c_ = 134 K for hydrostatic pressure *P* = 0 GPa. Therefore, the requirement for temperature condition of HgBa_2_Ca_2_Cu_3_O_8+δ_ is more easily satisfied in comparison with other superconducting materials in experiments. The London penetration depth is *λ*_0_ for *T*_*e*_ = 0 K. and the value is *λ*_0_ = 6.1 μm for HgBa_2_Ca_2_Cu_3_O_8+δ_.

Under hydrostatic pressure, the thickness of lattice element is modified by the strain formula

$$d_{a}(P)=d_{a0}[1-(S_{11}+2S_{12})P]$$ (3)

where d_a0_ is the thickness of element A with the value of *P* = 0 GPa, and for HgBa_2_Ca_2_Cu_3_O_8+δ_, the elastic constants are respectively given by S_11_ = 4.2×10^−3^ GPa^−1^ and S_12_ = −1.64×10^−3^ GPa^−1^ [20]. Without hydrostatic pressure, the value of refractive index of HgBa_2_Ca_2_Cu_3_O_8+δ_ is equal to n_a0_ = 0.9992 for the environment temperature value is set as *T*_*e*_ = 50 K. The initial thickness of A is suggested by d_a0_ = λ_c_/(4n_a0_) = 0.3878 μm, where λ_c_ = 1.55 μm is the central wavelength.

The semiconductor material of lattice element B is given by GaAs and its dielectric constant is a function of hydrostatic pressure (*P*) and circumstance temperature (*T*_*e*_)

$$\varepsilon_{b}(P,T_{e})=12.74e^{-1.73\times 10^{-3}P}e^{9.4\times 10^{-5}(T_{e}-75.6)},T_{e}<200K,$$ (4)

where *K* is temperature unit Kelvin [20]. Without applied hydrostatic pressure (*P* = 0 GPa), the thickness of lattice element B is denoted by d_b0_. Subsequently, the elastic constants of GaAs are listed by S_11_ = 1.16×10^−2^ GPa^−1^ and S_12_ = −3.7×10^−3^ GPa^−1^ with a nonzero hydrostatic pressure (*P* ≠ 0 GPa) [20]. The magnitude of elastic constants of GaAs is one order larger than that of HgBa_2_Ca_2_Cu_3_O_8+δ_, so the influence of hydrostatic pressure on the thickness of element A can be neglected in simulation. The refractive index of GaAs is n_b0_ = 3.565 with the conditions of *Te* = 50 K and *P* = 0 GPa. The thickness of element B is given by d_b0_ = λ_c_/(4n_b0_) = 0.1087 μm.

As a transverse magnetic wave is obliquely incident in the structure, we denote the incident light beam by the symbol *I*_*i*_, and *I*_*r*_ and *I*_*o*_ represent the reflected and transmitted light beams, respectively. The incident angle is set as *θ* = 10° and the spatially lateral shift of reflected beam is defined as Goos-Hänchen (GH) shift.

The defect superconductor PCs support a defect mode, of which the optical wave transmittance approaches to 1 and the reflectance is approximate to zero. The phase of reflected coefficient exists uncertainty at the zero point of reflectance. Consequently, giant GH shift of reflected beam is bound to induce around the defect mode since GH shift is proportional to the gradient of reflected coefficient phase curve.

## 3. GH shift depending on pressure and temperature

**Fig 2:**
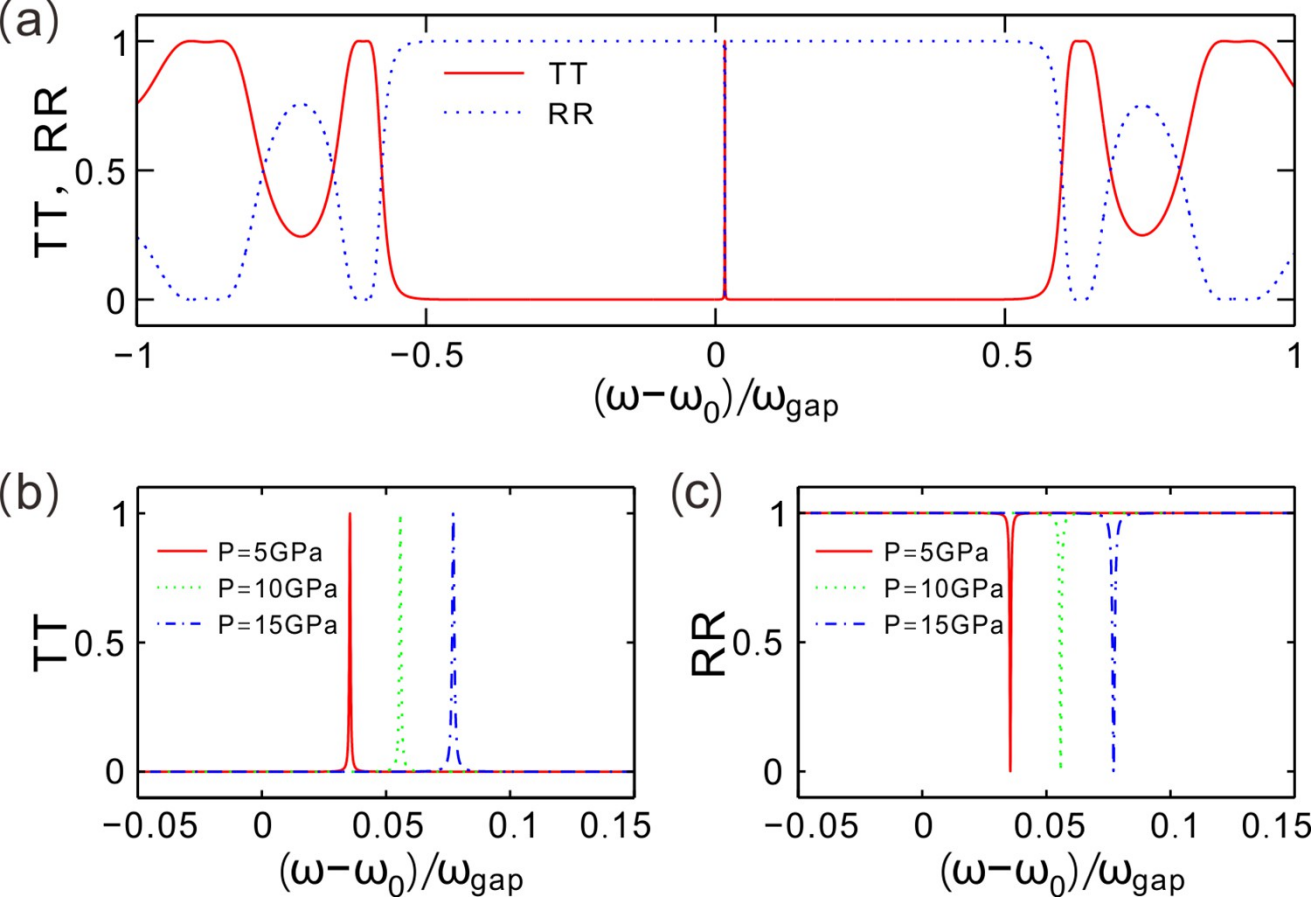
(**a**) Transmittance and reflectance of light waves changing with the normalized frequency for *P* = 0 GPa. (**b, c**) Transmittance and reflectance around the defect mode for different values of hydrostatic pressure, respectively. The environment temperature is set as *Te* = 50 K for (a)-(c).

Fig 2(A) gives the transmittance *TT* and reflectance *RR* of light waves. As the frequency of light waves changes, there is a bandgap arises in the optical wave spectrum of transmission. *TT* and *RR* are calculated by the transmission matrix method (TMM) [21]. The incident wave is transverse magnetic-polarized. The parameter of normalized frequency is denoted by (ω−ω_0_)/ω_gap_ and ω_gap_ = 4ω_0_arcsin│(n_a0_−n_b0_)/(n_a0_+n_b0_)|^2^/π, where *ω =* 2πc/λ, ω_0_ *=* 2πc/λ_c_, and c is the speed of light in free space. The presupposed environment temperature *T*_*e*_ = 50 K is lower than the critical temperature *T*_c_ = 134 K, then the superconducting material HgBa_2_Ca_2_Cu_3_O_8+δ_ is weak loss, which induces the transmittance approaching to *TT* ≈ 1, in the bandgap. Particularly, a resonant mode stemming from the central defective layer in PCs exists in middle of the bandgap. The defect mode is also a entirely transmitted mode, so the peak transmittance is *TT*_*p*_ ≈ 1. Superconductors are lossless for DC signals, while the loss in determined by the frequency of AC signals and the London penetration depth *λ*_L_ which relates to hydrostatic pressure or environment temperature as given in Eq (1). In out simulation, the superconductor HgBa_2_Ca_2_Cu_3_O_8+δ_ exists weak loss for AC light waves and the device dimension is only several times of the central wavelength, so the loss of light wave can be neglected. The reflectance of light *RR* form a complementary structure of the transmittance, satisfying *TT* + *RR* ≈ 1 for the concerned frequency section. The light waves have been reflected completely except for the defect mode.

For different values of hydrostatic pressure, Fig 2(B) demonstrates the transmittance of light around the defect mode of PCs. We choose three fixed hydrostatic pressure *P* = 5, 10 and 15 GPa to describe the properties of transmission spectra. One can see that the defect mode move towards higher frequencies as the hydrostatic pressure increases. The transmission peak values are *TT* ≈ 1 and the half width increases with the hydrostatic pressure. The central frequencies are (ω−ω_0_)/ω_gap_ = 0.0355, 0.0558 and 0.0771 for *P* = 5, 10 and 15 GPa, respectively

For the counterpart to transmittance, Fig 2(C) provides the reflectance of light around the three defect modes for *P* = 5, 10 and 15 GPa. There is a valley in each reflection spectrum and the minimum reflectance is equal to 0. Each zero point of reflection is exactly arising at the frequency of the corresponding transmission peaks. Therefore, the defect mode of light waves can be modulated through the hydrostatic pressure.

The critical temperature *T*_c_ = A_1_ + B_1_*P* + C_1_*P*^2^ is a function of hydrostatic pressure *P* and the London penetration depth *λ*_L_ is governed by *T*_c_ as shown in Eq (2). The dielectric constant of HgBa_2_Ca_2_Cu_3_O_8+δ_ is further determined by *λ*_L_ given in Eq (1). The value of refractive index of HgBa_2_Ca_2_Cu_3_O_8+δ_ decreases with the increase of hydrostatic pressure *P*. On the other hand, the thickness of slab of HgBa_2_Ca_2_Cu_3_O_8+δ_ also decreases slightly as hydrostatic pressure *P* increases. Based on the relation *d*_*a*_ = λ_c_/(4*n*_*a*_), one can deduce the result that the central wavelength λ_c_ decreases by turning up the value of *P*. Therefore, the defect mode of light waves, *viz*. The resonant peak of *TT*, or the valley of *RR*, moves to higher frequencies as hydrostatic pressure *P* increases.

**Fig 3:**
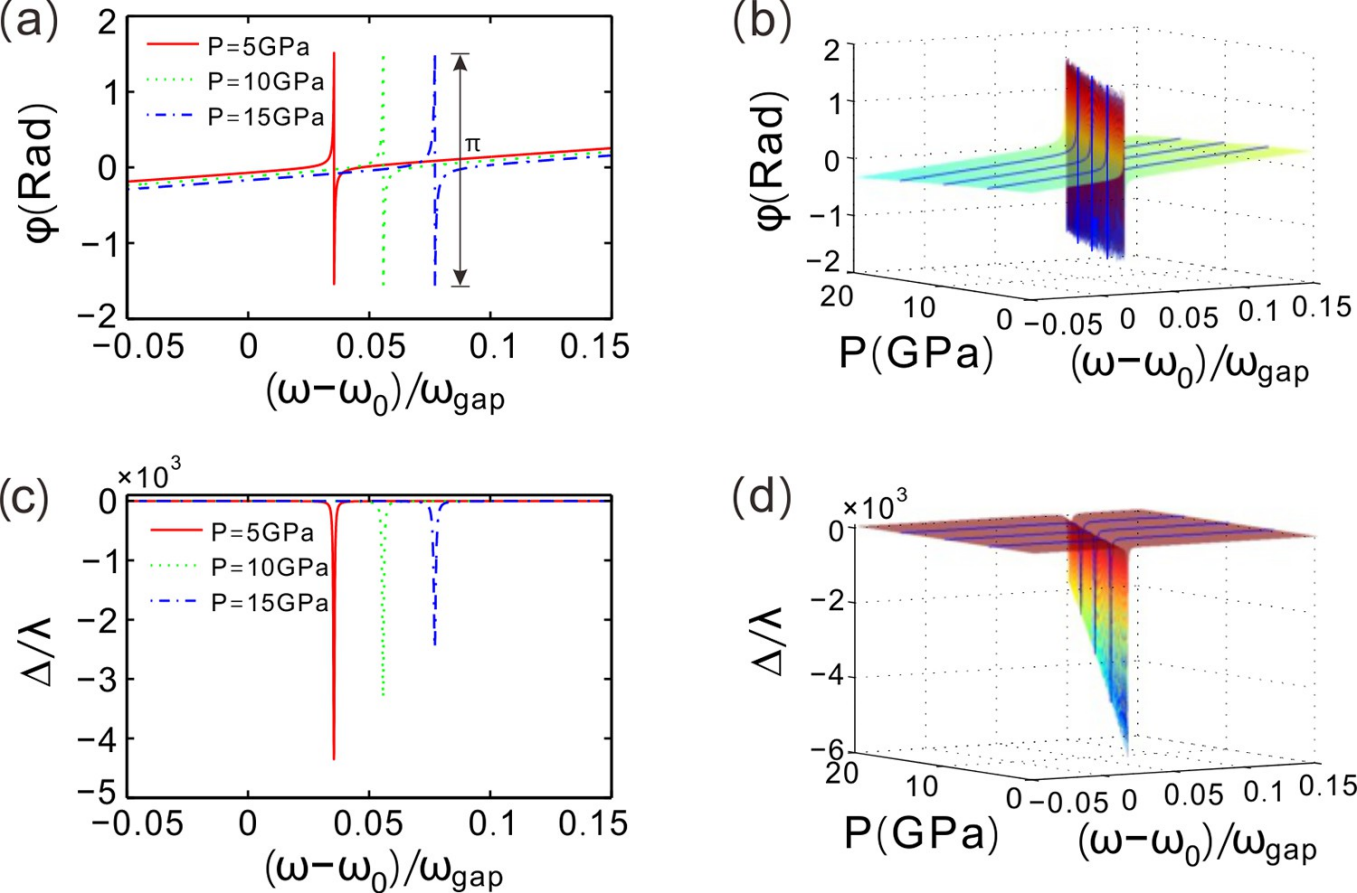
(**a, c**) Phase of reflection coefficient and GH shift of reflected beam changing with the frequency around the defect mode for different values of hydrostatic pressure, respectively. (**b, d**) Phase of reflection coefficient and GH shift in the parameter space composed of hydrostatic pressure and frequency, respectively. Environment temperature is given by *Te* = 50 K.

The reflection coefficient can be denoted by *r* = |*r*|exp(*iφ*), where |*r*| is the amplitude and *φ* is the complex angle. The structure, incident angle and environment temperature are fixed. For a given pressure value *P* = 5 GPa, the corresponding reflection coefficient phase abruptly jump at the defect mode (ω−ω_0_)/ω_gap_ = 0.0355 with a π change in phase as shown in Fig 3(A). Around the defect mode, the reflection coefficient phase sharply changes with the increase of the normalized frequency, and the slope of phase curve is extremely large. Then, giant GH shift of reflected beam may be induced around the defect mode. For *P* = 10 or 15 GPa, it also exists a π phase jumping at the defect mode in the corresponding phase curve. The phase changes acutely near (ω−ω_0_)/ω_gap_ = 0.0558 or 0.0771 as well. Great GH shift may also be induced around the defect mode and this lateral shift can be modulated by hydrostatic pressure.

Fig 3(B) demonstrates the reflection coefficient phase in the parameter space. The parameter space is composed of hydrostatic pressure and the normalized frequency. By turning up the value of hydrostatic pressure, the normalized frequency of the defect mode shows a blue-shift property. Therefore, the jump point of phase in parameter space can be regulated finely by hydrostatic pressure. The spatially lateral shift of reflected beam is proportional to the rate of change in reflection coefficient phase, so consequentially giant GH shift can be resulted around these defect modes as mentioned above.

Fig 3(C) gives the GH shift of reflected beam changing with the normalized frequency for different values of hydrostatic pressure. Around the defect mode, the GH shift is negative and the maximum GH shift of reflected beam is as high as −4.35×10^3^ times of incident wavelength λ at (ω−ω_0_)/ω_gap_ = 0.0355. For hydrostatic pressure *P* = 10 and 15 GPa, the maximum GH shifts are respectively −3.31×10^3^λ at (ω−ω_0_)/ω_gap_ = 0.0558 and −2.47×10^3^λ at (ω−ω_0_)/ω_gap_ = 0.0771. Obviously, the position of the maximum GH shift on the frequency-axis moves right and the valley shift value decreases as hydrostatic pressure increases.

Fig 3(D) gives the spatial GH shift of reflected beam in the parameter space in the parameter space. There is a ravine in the curved surface of GH shift. Of which the valley corresponds to the maximal negative GH shift for each given hydrostatic pressure. The quantity of the maximum GH shift and its location could be tuned by the hydrostatic pressure.

**Fig 4:**
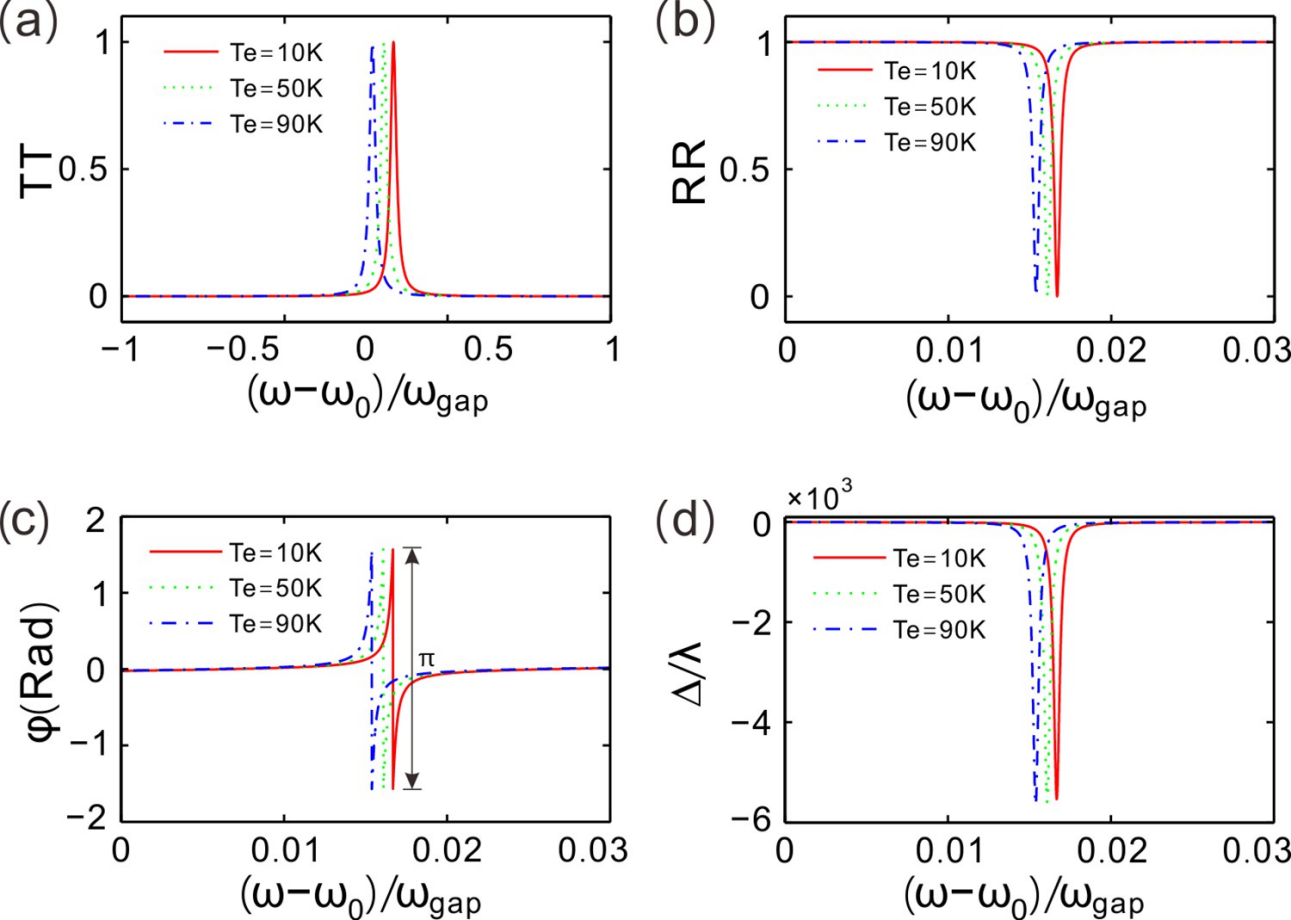
(**a, b**) Transmittance and reflectance of light waves around the defect modes for different values of environment temperature, respectively. (**c**) Reflection coefficient phase changing with the frequency near the defect mode for different values of environment temperature. (**d**) GH shift of reflected beam for different values of environment temperature. Hydrostatic pressure is set as *P* = 0 GPa and incident angle is *θ* = 10° for (**a**)-(**d**).

To investigate the influence of environment temperature on the optical properties of this photonic crystal, Fig 4(A) gives the transmission spectra for different values of environment temperature. Hydrostatic pressure is given by *P* = 0 GPa and the incident angle is fixed at *θ* = 10°. One can clearly observe that there is a photonic bandgap in each transmission spectrum and a transmission peak arises in the center of this bandgap. The transmission peak corresponds to the defect mode which is also a resonant mode. The three defect modes locate at (ω−ω_0_)/ω_gap_ = 0.0167, 0.0161 and 0.0154, respectively. The transmission peak, i.e. the defect mode, makes a red shift in wavelength as environment temperature rises. The transmittance slightly decreases with increase of environment temperature as well.

For a cluster of environment temperature, Fig 4(B) gives the counterparts of transmission spectra, *viz*. reflection spectra. It shows that there are three low-reflection modes in the center of the highly reflected bandgap for *T*_*e*_ = 10, 50 and 90 K, but the discrimination is not obvious enough for these defect modes. The reflection valley appears at the corresponding position at each transmission peak, and makes a red shift in wavelength as environment temperature increases as well. Meanwhile, the reflectance increases with increase of *Te*. This property manifests the defect mode could be modulated by environment temperature.

For the three given values of environment temperature *T*_*e*_ = 10, 50 and 90 K, Fig 4(C) exhibits the dependence of the phase of reflection coefficient on the normalized frequency. It shows that the reflection coefficient phase has a π change at the defect mode, around which the phase acutely varies as the normalize frequency changes. The zero reflectance at the defect mode results the uncertainty in reflection coefficient phase. The phase variation is more dramatic as the frequency get closer to the defect mode, which may induce great lateral shift of reflected beams.

Fig 4(D) describes GH shift changing with the normalized frequency for the three given values of environment temperature *T*_*e*_ = 10, 50 and 90 K. The GH shift is negative and at the defect mode the maximum GH shift can reach up to −5.53×10^3^*λ* at (ω−ω_0_)/ω_gap_ = 0.0167 for *T*_*e*_ = 10 K. The position of the maximum GH shift moves left on the frequency axis. The giant GH shift at the defect mode has been induced at the expense of zero reflectivity. GH shift exponentially decays as the frequency spreads from the defect mode to the right and left sides. Therefore, GH shift is sensitive to the incident wavelength (frequency) of light for a given value of *T*_*e*_, which can be utilized for highly sensitive wavelength sensors.

**Fig 5:**
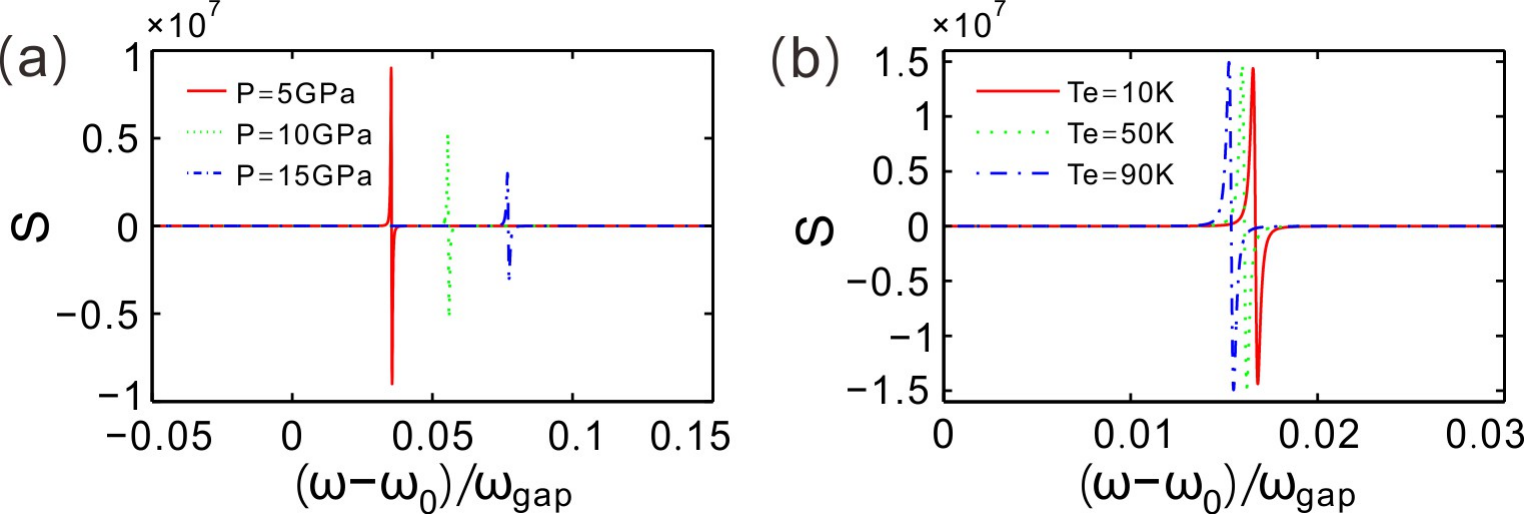
(**a**) Sensitivity coefficient of GH shift to incident wavelength near the defect mode for different values of hydrostatic pressure. (**b**) Sensitivity coefficient of GH shift to incident wavelength for different values of environment temperature. Enviroment temperature is set as *Te* = 50 K for (a). Hydrostatic pressure is set as *P* = 0 GPa for (**b**). Incident angle is given by *θ* = 10° for (**a**) and (**b**).

For different values of hydrostatic pressure *P* = 5, 10 and 15 GPa, Fig 5(A) gives the sensitivity coefficient of GH shift to the incident wavelength. The sensitivity coefficient is denoted by S which is changing with the normalized frequency. The value of S is positive and then becomes negative as the normalized frequency increases in each curve. The maximum of positive S is 9.005×10^6^ and the maximum of negaive S is −9.008×10^6^ for *P* = 5 GPa. For *P* = 10 and 15 GPa, the maxima of positive S are 5.298×10^6^ and 2.997×10^6^, respectively. The corresponding maxima of negative S are respectively −5.294×10^6^ and −2.998×10^6^, for *P* = 10 and 15 GPa. Therefore, the maximum of sensitivity coefficient of GH shift to the incident wavelength decreasese as the value of hydrostatic pressure increases.

Similarly, Fig 5(B) demonstrates the sensitivity coefficient of GH shift to the incident wavelength for different values of environment temperature. For *T*_*e*_ = 10, 50 and 90 K, the maxima of positive S are 1.438×10^7^, 1.467×10^7^ and 1.493×10^7^, respectively, while the corresponding maxima of negative S are respectively −1.438×10^7^, −1.467×10^7^ and −1.493×10^7^. The maximum of sensitivity coefficient of GH shift to the incident wavelength increasese as the value of environment temperature increases. The point of the maximum sensitivity coefficient in the frequency axis moves left by increasing environment temperature. Therefore, the sensitivity coefficient of GH shift to the incident wavelength could be regulated by hydrostatic pressure and environment temperature.

**Fig 6:**
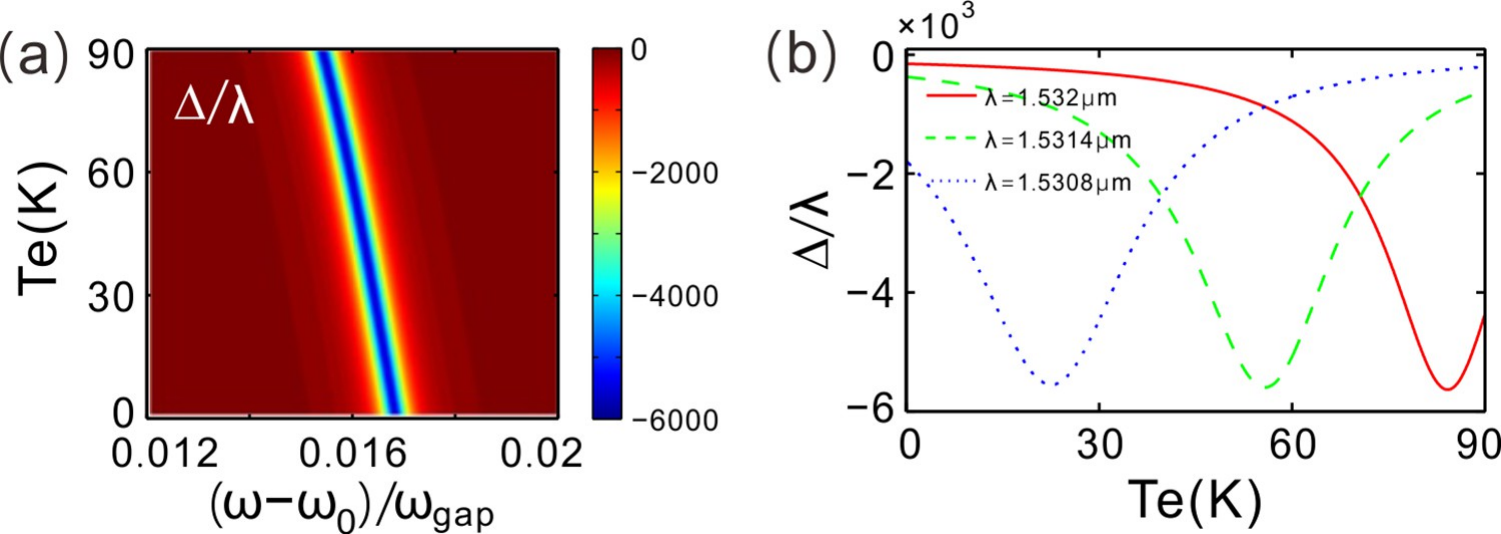
(**a**) GH shift in parameter space composed of environment temperature and normalized frequency. (**b**) GH shift changing with environment temperature.

Continuously modulating environment temperature and the normalized frequency, Fig 6(A) gives GH shift in the parameter space. There is a groove (the blue region) on the GH shift curved surface. GH shift is negative and the maximum of GH shift is as high as −10^3^λ. By increasing environment temperature, the corresponding normalized frequency of the maximum GH shift at the position in the parameter space moves to low frequencies. Otherwise, GH shift can be sensitive to environment temperature for a given normalize frequency, which is also corresponding to a fixed incident wavelength.

Fig 6(B) demonstrates the properties of GH shift changing with environment temperature for some different incident wavelengths. We here randomly choose three different incident wavelengths *λ* = 1.532, 1.5314 and 1.5308 μm. One can see that GH shift varies with environment temperature. GH shift is negative and there is a negatively maximum GH shift at *T*_*e*_ = 84.2 K. The maximum of GH shift is −5.63×10^3^*λ* for *λ* = 1.532 μm. For the other two wavelengths *λ* = 1.5314 and 1.5308 μm, the correspondingly maximum GH shifts are −5.6×10^3^*λ* at *T*_*e*_ = 55.8 K and −5.55×10^3^*λ* at *T*_*e*_ = 22.6 K, respectively. Therefore, the GH shift effect of the photonic structure can also be utilized for temperature sensors. To demonstrate this function, the incident wavelengths is set as *λ* = 1.532 and the incident angle is given by *θ* = 10°, then, the GH shift is −1.115×10^3^*λ* for environment temperature *T*_*e*_ = 60 K, The value of GH shift increase to −1.549×10^3^*λ* for *T*_*e*_ = 65 K and it further turns into −2.259×10^3^*λ* for *T*_*e*_ = 70 K. One can probe the location of reflected beam to determine the value of environment temperature based on these relation curves.

For light waves propagating, proximity effects in experiments at the interface between semiconductor layer and superconductor layer can be exactly equivalent to fluctuations in refractive index or thickness of materials in simulations. One can see that a change in hydrostatic pressure or environment temperature can result a fluctuation in the refractive index or thickness of semiconductor layer and superconductor layer. The fluctuation in parameters of refractive index or thickness only lead to the defect mode shift in optical spectra, but the defect mode still arises in the photonic bandgap. The giant GH shift can also be achieved since a remarkable GH effect originates from the abrupt change in reflection coefficient phase at the defect resonance state.

## 4. Conclusions

In conclusion, GH shift of reflected and transmitted beams are explored in the superconducting and semiconducting PCs. Superconductor and semiconductor alternatively arrange to form PCs and a defect locates at the center. The phases of reflection and transmission coefficients change dramatically around the defect mode and great negative GH shifts have been resulted by modulating environment temperature and hydrostatic pressure. The maximum negative GH shift can reach as high as −10^3^ times of the incident wavelength. Furthermore, GH shifts are extremely sensitive to environment temperature and hydrostatic pressure, so this study may be found an application for stationary pressure- or temperature light beams sensors in cryogenic environment.

## Supporting information

S1 Data (RAR)

## References

[1] Yallapragada V. J.; Ravishankar A. P.; Mulay G. L.; Agarwal G. S.; Achanta V. G. Observation of giant Goos-Hänchen and angular shifts at designed metasurfaces. Sci. Rep., 2016, 6, 19319. 26758471 10.1038/srep19319 PMC4725830

[2] Kong Q.; Shi H.; Shi J.; Chen Xi. Goos-Hänchen and Imbert-Fedorov shifts at gradient metasurfaces. Opt. Express, 2019, 27(9), 11902–11913. 31052739 10.1364/OE.27.011902

[3] Zhen W.; Deng D.; Guo J. Goos-Hänchen shifts of Gaussian beams reflected from surfaces coated with cross-anisotropic metasurfaces. Opt. Laser Technol., 2021, 135, 106679.

[4] Merano M.; Götte J. B.; Aiello A.; van Exter M. P.; Woerdman J. P. Goos-Hänchen shift for a rough metallic mirror. Opt. Express, 2009, 17(13), 10864–10870. 19550486 10.1364/oe.17.010864

[5] Merano M.; Aiello A.; ’t Hooft G. W.; van Exter M. P.; Eliel E. R.; Woerdman J. P. Observation of Goos-Hänchen shifts in metallic reflection. Opt. Express, 2007, 15(24), 15928–15934. 19550880 10.1364/oe.15.015928

[6] Zang M.; He T.; Zhang B.; Zhong L.; Shen J. Temperature-dependent Goos–Hänchen shift in the terahertz range. Opt. Commun., 2016, 370, 81–84.

[7] Goos F.; Hänchen H. Ein neuer und fundamentaler versuch zur totalreflexion. Ann. Phys., 1947, 436(7–8), 333–346.

[8] Wang L. G.; Zhu S. Y. Large positive and negative Goos-Hänchen shifts from a weakly absorbing left-handed slab. J. Appl. Phys., 2005, 98(4), 333.

[9] Guo H., Zhao D. Giant spatial Goos–Hänchen shifts in a non-hermitian dielectric slab sandwiched by graphene. Optik, 2021, 242, 167332.

[10] Cao Y., Fu Y., Zhou Q., Xu Y., Gao L., Chen H. Giant Goos-Hänchen shift induced by bounded states in optical PT-symmetric bilayer structures. Opt. Express, 2019, 27(6), 7857–7867. 31052613 10.1364/OE.27.007857

[11] Takeda H.; Yoshino K. Tunable photonic band schemes in two-dimensional photonic crystals composed of copper oxide high-temperature superconductors. Phys. Rev. B, 2003, 67(24), 245109.

[12] Hao J. J.; Gu K. D.; Xia L.; Liu Y. J.; Yang H. W. Research on low-temperature blood tissues detection biosensor based on one-dimensional superconducting photonic crystal. Commun. Nonlinear Sci., 2020, 89(20), 105299.

[13] Baraket Z.; Zaghdoudi J.; Kanzari M. Investigation of the 1D symmetrical linear graded superconductor-dielectric photonic crystals and its potential applications as an optimized low temperature sensors. Opt. Mater., 2017, 64, 147–151. doi: 10.1016/j.optmat.2016.12.005

[14] Yamamoto A.; Takeshita N.; Terakura C.; Tokura Y. High pressure effects revisited for the cuprate superconductor family with highest critical temperature. Nat. Commun., 2015, 6, 8990. doi: 10.1038/ncomms9990 26619829 PMC4686855

[15] Taya S. A.; Ramahi O. M.; Abutailkh M. A.; Doghmosh N.; Colak I. Investigation of bandgap properties in one-dimensional binary superconductor–dielectric photonic crystal: TE case. Indian J. Phys., 2022, 96, 2151–2160.

[16] Segovia-Chaves F.; Vinck-Posada H. Tuning of the defect mode in a 1d superconductor-semiconductor crystal with hydrostatic pressure dependent frequency of the transverse optical phonons. Physica C, 2019, 556(15), 7–13.

[17] Aly A. H.; Mohamed D.; Elsayed H. A.; Mehaney A. Fano resonance by means of the one-dimensional superconductor photonic crystals. J. Supercond. Nov. Magn., 2018, 31(12), 3827–3833.

[18] Xu B.; Zhao X.; Li G.; Zhang P.; Hua R. Large spatial Goos-Hänchen shifts from quasicrystals with graphene. Results Phys., 2020, 19, 103349.

[19] Segovia-Chaves F.; Vinck-Posada H. Effects of temperature, pressure and thickness on a one-dimensional Thue-Morse photonic crystal. Optik, 2020, 203, 163887.

[20] Segovia-Chaves F.; Vinck-Posada H. Transmittance spectrum of a superconductor-semiconductor quasiperiodic one-dimensional photonic crystal. Physica C, 2019, 563, 10–15.

[21] Fang M.; Wang Y.; Zhang P.; Xu H.; Zhao D. Multiple exceptional points in APT–symmetric Cantor multilayers. Crystals, 2023, 13(2), 197.

